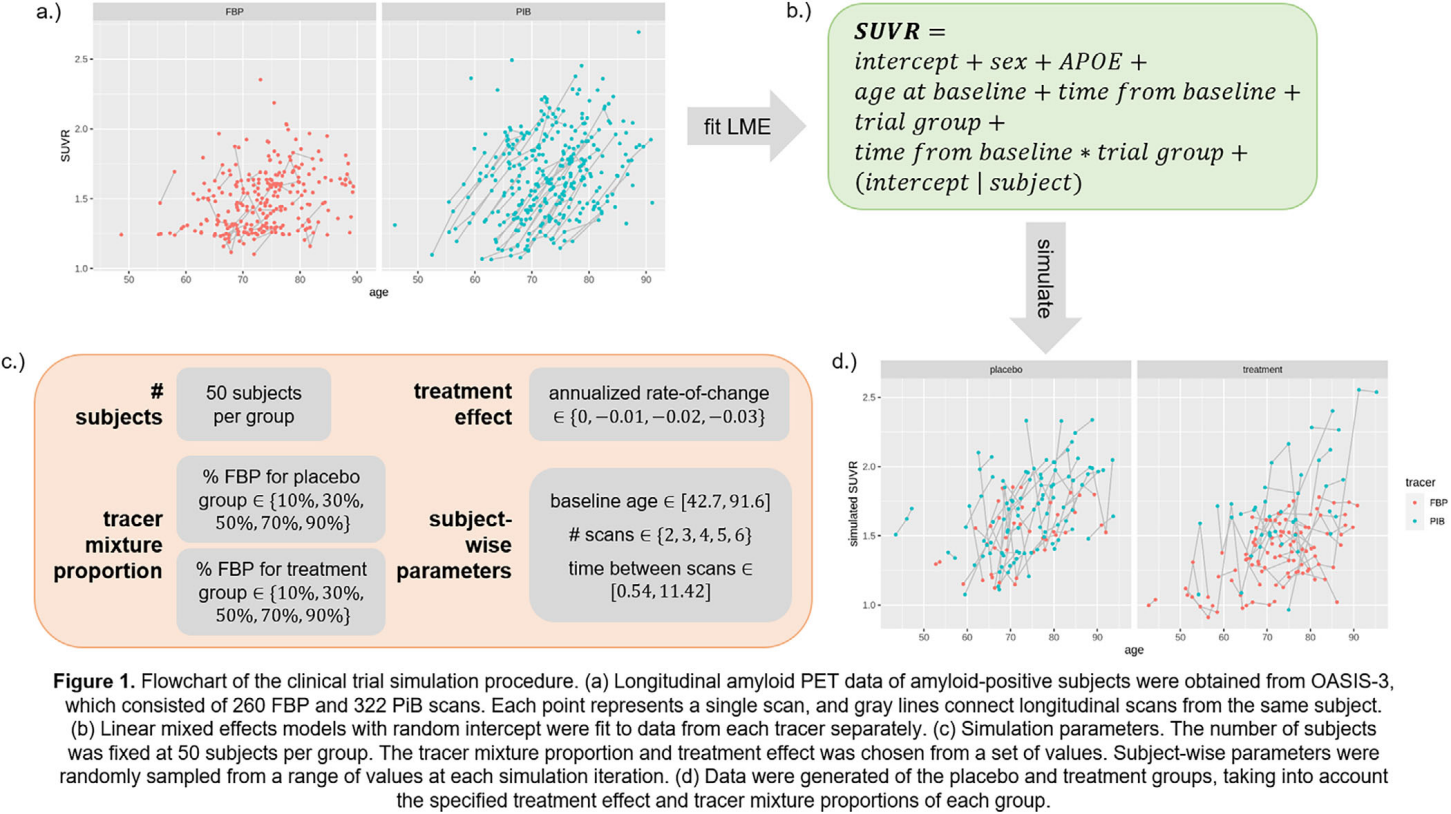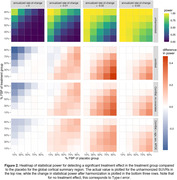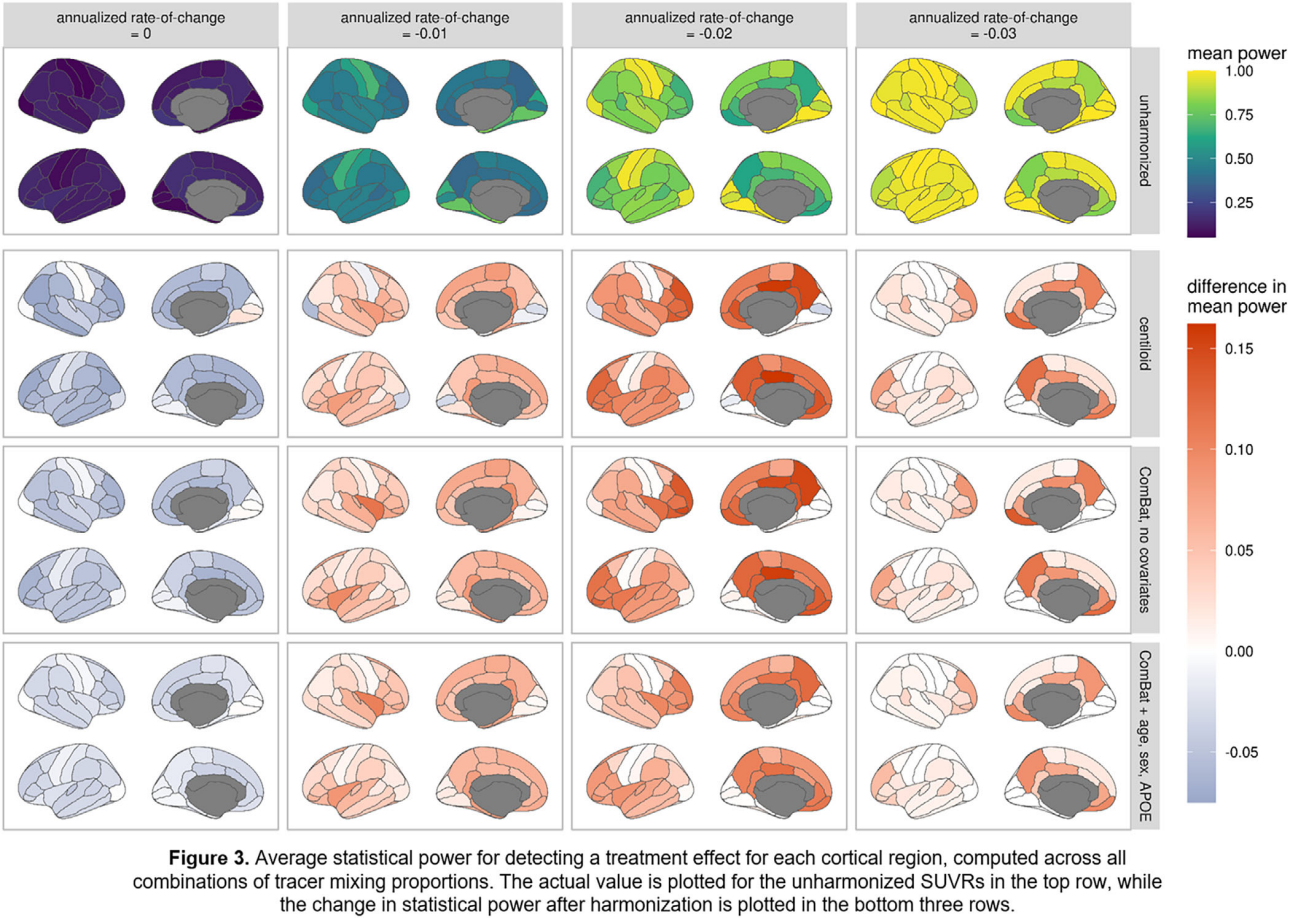# Harmonization of amyloid PET radiotracers using ComBat and its influence on detecting treatment effects in a simulated clinical trial

**DOI:** 10.1002/alz.094115

**Published:** 2025-01-09

**Authors:** Braden Yang, Tom Earnest, Sayantan Kumar, Brian A. Gordon, Aristeidis Sotiras

**Affiliations:** ^1^ Mallinckrodt Institute of Radiology, Washington University School of Medicine in St Louis, St Louis, MO USA; ^2^ Mallinckrodt Institute of Radiology, Washington University in St. Louis, St. Louis, MO USA; ^3^ Institute for Informatics, Washington University, St. Louis, MO USA

## Abstract

**Background:**

Differences in amyloid PET radiotracer pharmacokinetics and binding properties lead to discrepancies in amyloid uptake measurements, which may adversely affect the statistical power of clinical trials that utilize multiple tracers to track brain amyloid deposition. To address this, Centiloid was developed for standardizing global amyloid SUVRs across tracers to a common scale. Alternatively, ComBat is a technique for harmonizing batch effects while preserving variations from biologically‐relevant covariates. Unlike Centiloid, ComBat is entirely data‐driven, does not require a calibration process, and can be applied to regional SUVRs. However, it has not been validated for amyloid PET. Here, we evaluate whether ComBat improves detection of treatment effects in a simulated clinical trial.

**Method:**

365 amyloid‐positive subjects were identified from the OASIS‐3 dataset, from which 322 Pittsburgh compound B (PiB) and 260 18F‐Florbetapir (FBP) PET scans were selected. SUVRs of the global cortical region and 82 FreeSurfer regions were computed from each scan using the PET Unified Pipeline. Linear mixed effects (LME) models were fitted on each tracer separately and used to simulate longitudinal SUVRs of placebo and treatment groups in a hypothetical clinical trial (Figure 1). A negative rate‐of‐change was introduced to the treatment group to mimic a treatment effect. Additionally, tracer mixing proportions were varied within each group. Centiloid and ComBat were applied to simulated data to harmonize across tracers. A separate LME model was fitted to test for significant differences in SUVR rate‐of‐change between groups. Power was estimated as the proportion of significant findings across 1000 simulations.

**Result:**

After harmonization with either Centiloid or ComBat with no covariates, an increase in power was observed in the presence of a treatment effect, and a decrease in Type‐I error was observed for no treatment effect (Figure 2). These changes were most prominent in cases where groups exhibited differing tracer mixing proportions. Similar patterns were observed for regional SUVRs (Figure 3).

**Conclusion:**

We demonstrated that tracer harmonization is important for improving power in the presence of a treatment effect and reducing Type‐I error in its absence. ComBat performs comparably to Centiloid in harmonizing amyloid radiotracers in the context of a clinical trial.